# Does the Structured Operational Research and Training Initiative (SORT IT) continue to influence health policy and/or practice?

**DOI:** 10.1080/16549716.2018.1500762

**Published:** 2018-08-06

**Authors:** Jaya Prasad Tripathy, Ajay MV Kumar, Nathalie Guillerm, Selma Dar Berger, Karen Bissell, Anthony Reid, Rony Zachariah, Andrew Ramsay, Anthony D Harries

**Affiliations:** a International Union Against Tuberculosis and Lung Disease, South-East Asia Office, New Delhi, India; b International Union Against Tuberculosis and Lung Disease, Paris, France; c Médecins Sans Frontières, Medical Department, Operational Centre Brussels, Luxembourg; d Special Programme for Research and Training in Tropical Diseases, World Health Organization, Geneva, Switzerland; e School of Medicine, University of St Andrews, Fife, UK; f London School of Hygiene & Tropical Medicine, London, UK

**Keywords:** Operational research, policy, The Union, Médecins Sans Frontières, TDR, SORT IT

## Abstract

**Background**: The Structured Operational Research and Training Initiative (SORT IT) is a successful model of integrated operational research and capacity building with about 90% of participants completing the training and publishing in scientific journals.

**Objective**: The study aims at assessing the influence of research papers from six SORT IT courses conducted between April 2014 and January 2015 on policy and/or practice.

**Methods**: This was a cross-sectional mixed-method study involving e-mail based, self-administered questionnaires sent to course participants coupled with telephone/Skype/in-person responses from participants, senior facilitators and local co-authors of course papers. A descriptive content analysis was performed to generate themes.

**Results**: Of 71 participants, 67 (94%) completed the course. A total of 67 papers (original research) were submitted for publication, of which 61 (91%) were published or were in press at the censor date (31 December 2016). Among the 67 eligible participants, 65 (97%) responded to the questionnaire. Of the latter, 43 (66%) research papers were self-reported to have contributed to a change in policy and/or practice by the course participants: 38 to a change in government policy or practice (26 at the national level, six at the subnational level and six at the local/hospital level); four to a change in organisational policy or practice; and one study fostered global policy development.

**Conclusion**: Nearly two-thirds of SORT IT course papers contributed to a change in policy and/or practice as reported by the participants. Identifying the actual linkage of research to policy/practice change requires more robust methodology, in-depth assessment and independent validation of the reported change with all concerned stakeholders.

## Background

Operational research (OR), from a public health perspective, has been defined as the search for knowledge on interventions, strategies or tools that can enhance the quality, coverage, effectiveness or performance of the health system, specific health services or disease control programmes in which the research is being conducted [1]. OR is gaining recognition and importance in public health, guiding national research agendas and programme implementation to achieve desired results [1,2]. Despite this, the implementation of OR is poor, especially in low- and middle-income countries (LMICs) where it has a vital role to play. One of the main reasons for this short-coming is the lack of capacity in conducting OR [1,3]. The International Union Against Tuberculosis and Lung Disease (The Union), Médecins Sans Frontières (MSF) and the Special Programme for Research and Training in Tropical Diseases (TDR) at the World Health Organization (WHO), have pioneered a programme of integrated OR and capacity building known as the Structured Operational Research and Training Initiative (SORT IT). The aim of the SORT IT programme is to teach the practical skills of conducting and publishing research, and to impact policy and/or practice. The end expected result is improved health care delivery and programme performance [4].

Measuring the impact on policy and/or practice is a crucial element in assessing the success of OR conducted through the SORT IT programme. To achieve this, a systematic follow-up is done 18 months after course completion to assess whether research conducted by the course participants has had any impact on policy and/or practice. Two previous studies revealed 56% and 74% of the course publications had contributed to changes in policy and/or practice within that timeframe [5,6]. In the meantime, scale-up of SORT IT has continued globally; new geographic regions have been included and there is a deliberate effort to decentralize training to regional and national levels. As policy and/or practice change may be affected by the above factors, assessing if new SORT IT courses continue to influence change in policy and/or practice is vital. In this paper we describe the self-reported contribution to policy and/or practice from six SORT IT courses completed between April 2014 and January 2015.

## Methods

### Study design

This was a mixed-methods study with a cross-sectional quantitative and a descriptive qualitative design.

### Setting

The structure and organization of the SORT IT courses have been described elsewhere [4]. Briefly, the course is conducted over a period of 10–12 months, and there are three modules: module 1 (OR protocol development); module 2 (data capture and analysis); and module 3 (paper writing), with four clearly defined milestones and expected outputs (Table 1). A maximum of 12 participants are selected per course. Individuals working in routine programme settings are preferred, with particular attention placed on the relevance of the OR question and use of routinely collected programme data. Participants receive a full scholarship, which includes course fee, accommodation and full meals, transport, and per-diems. Each module takes around 6–7 days and includes formal lectures, one-to-one mentoring of participants and group plenary sessions. The milestones have to be achieved for participants to remain on the course. Failure to meet any of the first three milestones results in non-eligibility of the participant to attend module 3, while failure to meet the final milestone (i.e. submission of the final paper to a peer-reviewed journal within 4 weeks of completion of module 3) results in the participant being declared a ‘course failure’.

**Table 1.** Milestones for SORT IT operational research and capacity-building programmes in Europe, Africa, Asia and the South Pacific starting from April 2014 to January 2015^a^.

Milestone	Details of the milestone
Milestone 1	Submission of the research protocol and the completed ethics application form to the course coordinator and ethics committee within 3 weeks of the end of module 1.
Milestone 2	Submission of data documentation sheet, EpiData triplet files (qes, rec and chk files) and dummy tables (indicating plan of analysis) to the course coordinator within 2 weeks of the end of module 2.
Milestone 3	Submission of proof of data collection to their respective facilitators and course coordinator 6 weeks before start of module 3.
Milestone 4	Submission of a manuscript to a peer-reviewed journal within 4 weeks of the end of module 3: copy of the submitted paper and email acknowledgement of receipt of the submitted paper by the journal both to be sent to the course coordinator and the ethics committee.

^a^This includes six courses in India (1), Nepal (1), Luxembourg (1), Fiji (1), Ethiopia (1) and Estonia (1)

Although these courses revolve around conducting and publishing OR within a certain time frame, there is a considerable focus on translating the evidence generated into policy and/or practice. On the very first day of the course there is a lecture on ‘Translating OR into policy and practice’ which discusses enablers and challenges to evidence translation, case studies on research impacting policy and/or practice, and what worked and what did not. The importance of choosing a policy-relevant research question, identifying and engaging policy makers/influencers from the beginning, and effective dissemination of research findings are all strongly emphasized early on in module 1.

### Study participants

The study population included all participants and senior facilitators of six SORT IT courses started between April 2014 and January 2015. The courses were run in Chennai, India; Kathmandu, Nepal; Luxembourg city, Luxembourg; Addis Ababa, Ethiopia; Nadi, Fiji; and Tallinn, Estonia.

### Sources of data, variables and data collection

Self-administered, structured questionnaires were e-mailed to the course participants 18 months after course completion and responses were emailed back to the Union headquarters in Paris, France. The questionnaire included closed ended quantitative and open ended qualitative variables. The quantitative variables included demographic details of the participant, affiliation and designation, details about the OR project s/he was involved in during the course, and its publication status. The qualitative variables included an open ended question assessing the effect of the research project on policy and/or practice. If the research was reported to have affected policy/practice change, the participants were contacted and a detailed explanation was sought, and if the research was not reported to affect policy/practice change, reasons were asked. Wherever required, senior facilitators and other local co-authors who were involved in the study, and other relevant stakeholders, were contacted over the telephone or Skype or in-person for further clarifications and to confirm the facts presented by the participants, and their responses were transcribed. The effect of any research project was considered ‘yes’ if it contributed to any change in policy and/or practice or scale up of an existing intervention at any level, be it national, subnational, local or organizational. Contribution to change in policy and/or practice was based on self-reports backed by documentary evidence and validated by other local or senior co-authors who were involved in the study. In order to maximize objectivity, the final decision about whether policy and/or practice change occurred was taken through consensus by a team of co-authors (JPT, AMVK, RZ, ADH). We took a conservative approach in determining whether a study contributed to the policy/practice – meaning. if there was any doubt, the study was considered not to have contributed to policy and/or practice.

The impact was assessed at global, government (which includes national, subnational or local/public hospital level) and organizational level, which includes non-governmental organizations (NGOs) and private sector hospitals.

### Data entry and analysis

The data (both quantitative and qualitative) were captured in an MS Excel format. Quantitative variables were summarized using proportions. For qualitative data, a descriptive content analysis of responses was performed. The content was analysed manually by two authors (JPT, AK), who are experienced in qualitative research methods. Themes were generated in consensus using standard procedures [7]. Any disagreement between the two authors was resolved by consensus after consultation with two senior authors (ADH, RZ). The following broad themes emerged from the responses: changes in government policy and/or practice (subdivided into national, subnational and local/hospital level depending on the level of impact) and changes in organizational policy and/or practice (when decisions were made by an organization such as the MSF or The Union to improve their performance). Themes were generated by two authors (JPT, AK) together to remove subjective bias and enhance interpretive credibility. The participants/senior facilitators were contacted again through e-mail/Skype/telephone in case any clarification was required.

### Ethics

Ethics approval for the study was obtained from the Ethics Advisory Group of The International Union Against Tuberculosis and Lung Disease (The Union), Paris, France. Since the 18 month assessment is performed routinely as part of the SORT IT programmes, the ethics advisory group waived the need for written informed consent for questionnaires. Participants were free not to respond to the questionnaires. Verbal informed consent of individuals who were interviewed was obtained.

## Results

### Quantitative findings

#### Characteristics of SORT IT participants

Of 71 (45% female) participants in the six courses, 67 (94%) successfully completed the course and submitted a paper each to a peer-reviewed scientific journal. Among 67 eligible participants, 65 (97%) responded to the questionnaire. The participants came from 36 countries across four continents (26 from Asia among which 12 were from India, 15 from Africa, 12 from the South Pacific, 13 from Europe and one from Latin America).

The majority of the participants were medical doctors (29), followed by public health specialists (14), research officers/programme managers (7), nurses (7), monitoring and evaluation officers (5), data managers/biostatisticians (2) and other paramedical staff (3). Most of them were working in the government sector (20), followed by NGOs (17), academia (9), hospitals (8), WHO (7) and research institutes (6).

#### Manuscript submissions, publications and self-reported changes in policy and/or practice

A total of 67 papers (original research) were submitted for publication, of which 61 (91%) were published or in press as of December 2016. The topics covered included 36 (54%) in tuberculosis (TB), 10 (15%) in non-communicable diseases, six (9%) in human immunodeficiency virus (HIV) infection, three (4%) in maternal and child health, three (4%) in neglected tropical diseases and nine (14%) in other subject areas (antibiotic prescribing, nosocomial infections, drug resistance, and other issues related to clinical care, health services and health systems).

**Figure 1.**
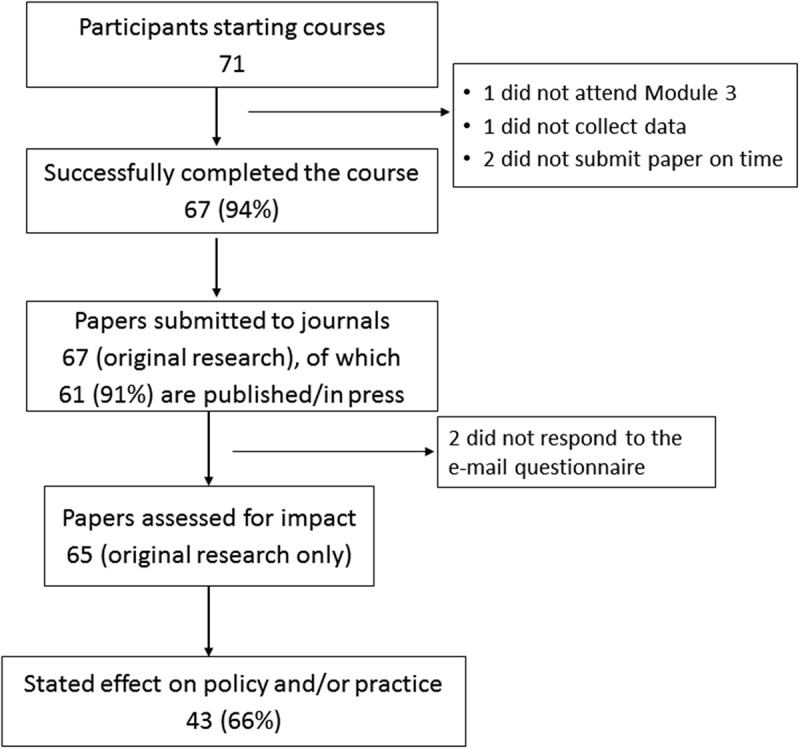
Programme outputs from six completed SORT IT courses conducted in Europe, Africa, Asia and the South Pacific during 2014–2015. Outputs from six completed SORT IT operational research courses run in Europe, Africa, Asia and the South Pacific during 2014–2015. The six courses were run in Chennai, India (*n* = 1); Tallinn, Estonia (*n* = 1); Luxembourg (*n* = 1); Addis Ababa, Ethiopia (*n* = 1); Nadi, Fiji (*n* = 1) and Kathmandu, Nepal (*n* = 1).SORT IT = Structured Operational Research and Training Initiative; NGO = non-governmental organisation.

Of the 65 papers assessed, 43 (66%) reportedly contributed to a change in policy and/or practice (Figure 1). Of the 43 papers that reported an impact, 38 contributed to a change in government policy or practice (26 at the national level, six at the subnational level and six at the local/public hospital level), four supported a change in organizational (NGOs, including private sector hospitals) policy and/or practice and one study contributed to global policy development. The changes could be classified into the following major themes: new policy decisions (*n* = 13); incremental changes in the existing policy strengthening the practice (*n* = 5); redrafting of national programme guidelines or clinical management protocols (*n* = 3); improved programme monitoring and reporting (*n* = 10); strengthening of information/surveillance systems or patient databases (*n* = 5); and strengthening of existing evidence leading to scale up of existing interventions (*n* = 7).

Table 2 gives examples of original research studies conducted within the SORT IT courses and their reported effect on policy and/or practice at national/subnational, local/hospital and organizational level. Other examples in varied contexts are given below with verbatim quotes from the course participants. All the examples given below and the citations provided are from the studies undertaken by the participants of the SORT IT programme.

**Table 2.** Examples of operational research and their effect on policy and/or practice from six completed courses during 2014–2015.

First author and country	Study description	Main findings and recommendations	Effect on policy and practice
**Change in national policy and/or practice**			
Narayan N. et al. (2014), Fiji [27]	A retrospective cohort study of all TB patients registered with the National Tuberculosis Programme (NTP) in Fiji between January 2011 and June 2013. To determine anti-tuberculosis treatment outcomes, stratified by method of treatment supervision (i.e. self-administered treatment [SAT] versus supervised treatment).	TB treatment outcomes were more likely to be successful in patients who were supervised than in SAT patients.	*‘TB patients are now not given an option of self-supervision. They are rather assigned to a DOT provider supported by the Ministry of Health.’*
Ciobanu A. et al. (2014), Moldova [28]	Retrospective cohort study using data from the national electronic patient database and incentive registers. The study objective was to compare treatment outcomes among new drug-susceptible TB patients registered for treatment before 2008 and after the 2011 introduction of incentives.	Provision of incentives to TB patients significantly improved treatment success rates and needs to continue.	*‘The published study was the basis for the National Tuberculosis Program to mobilize financial sources both from the national budget sources as well as the Global Fund to fund incentives for TB patients.’*
Mahajan R. et al. (2015), India [29]	This retrospective analysis included all patients with confirmed HIV-visceral leishmaniasis (VL) coinfection receiving combination treatment for VL at a MSF treatment centre between 2012 and 2014.	Combination therapy appeared to be well tolerated, safe and effective, and may be considered as an option for treatment of VL in HIV co-infected patients. Not initiating ART was associated with increased mortality.	*‘The study results strengthened emerging evidence that typical VL should be considered as a clear entry criterion to the stage IV definition of HIV and also support the need to offer Provider Initiated Counselling and Testing (PICT) to all patients diagnosed with VL. In the core group meeting between national programs for VL and HIV/AIDS, consensus was made on developing clearer recommendations and convergence between WHO ART guidelines and VL guidelines. Furthermore MSF is planning to do a clinical trial in collaboration with national health authorities to generate further robust evidence for best treatment options for HIV-VL co-infected patients in India.’*
Kyaw N. et al. (2015), Myanmar [30]	The study used routinely collected programme data on all patients aged ≥ 15 years starting first-line tenofovir-based ART between 2012 and 2013. Creatinine clearance was assessed at base line and 6 monthly and incidence of renal dysfunction was calculated.	The low incidence of renal toxicity in the patient cohort suggests that routine assessment of creatinine clearance may not be needed and could be targeted to high risk groups if resources permit.	*‘The national program adopted the alternative low cost screening method (urine protein testing) for screening of renal dysfunction in patients taking tenofovir and now uses the creatinine test only in high risk patients.’*
**Contributed to existing evidence leading to scale up of interventions**			
Dave P. et al. (2015), India [31]	This was a retrospective cohort study involving a review of electronic patient records maintained routinely under the National Tuberculosis Programme, Gujarat, India in 2013.	Offering rapid drug susceptibility testing (DST) at diagnosis improved the treatment outcomes among patients with pulmonary tuberculosis.	*‘The study findings strengthened the evidence of the effect of rapid DST at diagnosis on treatment outcomes of TB patients, which reinforced the national policy and has contributed to scale up of rapid DST facilities in the country.’*
**Change in policy and/or practice at subnational level**			
Shewade H. et al. (2014), India [32]	Mixed-methods study, quantitative component consisted of retrospective cohort study reviewing records of all presumptive MDR-TB patients between October 2012 and September 2013. The qualitative component included in-depth interviews with key informants involved in programmatic management of drug-resistant tuberculosis services.	High pre-diagnostic and pre-treatment attrition with 46% and 29%, respectively. Improving mechanisms for tracking patients referred for culture, developing a mechanism of sputum transport and training of health staff were recommended.	*‘All the recommendations proposed were implemented by the state authorities. Pre-diagnostic and pre-treatment attrition were reduced to 24% and 0%, respectively.’*
Repetto E.C. et al. (2015), Italy [33]	Retrospective cohort study involving review of patient data in an outpatient unit providing care for Chagas disease.	Among 210 patients eligible for treatment, 49% were lost-to-follow-up before treatment. The median delay from diagnosis to treatment was 4 months. Among 108 patients started on treatment only 58% completed treatment. The high prevalence of the disease and significant drop-outs along the care pathway require better access to care.	*‘In Bergamo, the local MoH started a structured OPD care unit for Chagas disease.’*
**Local change in policy and/or practice**			
Ganga Devi et al. (2015), India [34]	Retrospective cohort study reviewing records of all HIV-infected children (aged < 15 years) registered from 2005 to 2012 at an ART centre in Madurai, South India. The study aimed to assess the proportion of loss-to-follow up (LTFU) among children in pre-ART care.	LTFU was alarmingly high (63%), indicating poor clinical and programmatic monitoring among children in pre-ART care. A system for active tracing of those missing with intensified supervision and monitoring, along with further qualitative research to explore the reasons for such high rates of LTFU was recommended.	*‘This study proved to be an effective advocacy tool for intensification of supervision and monitoring of children on pre-ART care eventually leading to the introduction of an indicator to monitor pre-ART LTFU in the monthly reports. It also led to the conception of a similar study in the whole state of Tamil Nadu, India to document LTFU among children on pre-ART.’*
**Hospital level change in policy and/or practice**			
Bajis S. et al. (2014), Afghanistan [35]	Cross-sectional study, using routinely collected hospital data (analysis of prescriptions) to assess antibiotic prescribing practices in the out-patient department of a district hospital in Afghanistan.	Rates of antibiotic prescriptions for out-patients were high, double the WHO recommendation of 30%. Inappropriate prescriptions for specific conditions may have occurred. This study suggests that knowledge about context-specific determinants of antibiotic prescribing is a first step towards promoting rational prescribing practices in such settings.	*‘The study resulted in: a) a qualitative research looking at perceptions and attitudes among practitioners and patients towards antibiotic use; b) emphasis on education of staff, implementing follow up of antibiotic prescription for adherence; c) prescription forms being modified and merged with the medical record system; d) new doctors hired to work on rational prescribing of antibiotics.’*
Nanjebe D. et al.(unpublished), Uganda [36]	A retrospective study was conducted using routinely-collected data, evaluating how specimens for culture and sensitivity testing (C&S) were obtained, handled and used by clinicians to treat opportunistic infections (OIs) in patients with HIV in Mbarara Regional Referral Hospital, 2010–2013.	Only a small proportion of cultures resulted in a report filed in patients’ charts influencing clinicians’ antibiotic choices. This is important because performing C&S testing consumes laboratory resources and yet seems to have little effect on quality of care and choice of treatment.	*‘The study findings were presented to the hospital administrators and other key policy makers. This led to the designing of a database/information system for the hospital that will improve results communication between the laboratories and the hospital.’*
**Organizational change in policy and/or practice**			
Nsabuwera V. et al (2015), Rwanda [37]	The present study aimed to assess the changes in food access and consumption at the household level after 1 year implementation of an integrated food security intervention in three rural districts of Rwanda.	The study demonstrated that an integrated food security intervention, implemented in a setting of extreme poverty, was associated with considerable improvements in household food security.	*‘The study has influenced the scale up of the intervention package within the organization, from three health centre catchments to six.’*

DOT = Directly Observed Treatment; ART = Anti-Retroviral Therapy; MSF = Médecins Sans Frontières; MDR-TB = Multidrug Resistant Tuberculosis; HIV = Human Immunodeficiency Virus; AIDS = Acquired Immunodeficiency Syndrome.

Sentences in italics are verbatim quotes by the course participants about the impact of their research on policy and/or practice.

### Qualitative findings

#### Self-reported contribution to national policy and/or practice change

There were several examples of contribution to change in national policy/practice. Alikhanova et al. did the first national survey of anti-tuberculosis drug resistance in Azerbaijan and demonstrated a clear need for third-line anti-tuberculosis medicines [8]. The results of the study contributed to a new policy decision by the Ministry of Health. She [the author] said:

*Based on the results of this study, the Ministry of Health took a decision to procure TB medicines, which were not used before in the country like bedaquiline, linezolid, imipenem, cilastatin etc.*

A study by Atia et al. in Sudan generated evidence to influence changes in the national management protocol for visceral leishmaniasis (VL) and monitoring of adverse effects of the first-line VL treatment regimen [9]:

*Yes, now the first line for visceral leishmaniasis treatment (sodium stibogluconate/paromomycin) is under focus and the new national protocol (2016) addressed the study recommendations in term of uses of this regimen for relapse and in elderly; the VL programme added a pharmaco-vigilance component in the new VL patient card to monitor this regimen; use of new drugs like miltefosine and amphotericin-B is now promoted.*

A countrywide evaluation of decentralized TB clinics in Armenia [10] showed that some clinics performed very poorly in terms of TB case notifications and treatment success which contributed to a major policy decision:

*The research evaluated TB outpatient services in decentralised facilities and confirmed that 12 outpatient services have no efficiency in terms of TB detection and treatment. As a result these 12 TB outpatient services were optimised and merged with the nearest TB outpatient services, improving the financial and service delivery capacity of the joint TB outpatient services.*

A study on adverse drug events among a nationwide cohort of multi-drug resistant tuberculosis (MDR-TB) patients in Nigeria [11] impacted practice at the national level:

*The National Tuberculosis Programme used the findings of the study to mobilize greater focus on pharmacovigilance activities, which include detection, reporting and management of adverse drug reactions among patients with MDRTB. Prior to the study, Nigeria had no documented evidence of the most prevalent adverse reactions of second line anti-tuberculosis medications. This limited government efforts at drug regulatory activities as well as managing the affected patients. But with the findings of the study, Nigerian health workers now know the Adverse Drug Reactions to expect when people living with the MDRTB are placed on therapy.*

Some studies had an impact on the routine monitoring mechanism or recording and reporting system, such as those in Fiji [12,13] which contributed to improved reporting and recording of information of patients especially treatment outcomes of tuberculosis patients. A participant from Fiji said:

*Yes, there was a need to correctly carry out data entry especially when recording the treatment outcome and this was put into practice immediately after the research results were disseminated.*

#### Self-reported contribution to change in policy and/or practice at organizational level

Some research projects contributed to a change in organizational level policy or practice. In a project run by the MSF in Kibera, Kenya, a study by Some et al. showed that nurses were able to adhere to protocols for managing stable non-communicable disease (NCD) patients based on a clear standardized protocol [14]. This contributed to a major revision in the Kibera project guidelines which changed the way NCD patients are managed in those facilities. MSF also plans to implement the recommendations from this study in a proposed new project in a rural setting in Kenya:

*The study results led to the revision of patient flow in the clinics which enabled the nurses to see and manage stable NCD patients who initially had to see the doctor.*

#### Self-reported contribution to hospital level policy and/or practice change

A study in a naturopathy hospital in South India [15] had a profound impact on the research environment within the facility. Following this study, a new study was initiated addressing the limitations of the earlier study with a longer follow-up and a control group. A new department of research has been set up within the hospital with newly recruited staff. Other initiatives include capacity building of the existing staff, renaming of the hospital by including the phrase ‘research centre’, constituting an ethics committee and enhancing the infrastructure to conduct research. The hospital information system has been restructured to efficiently capture and store data from patient records to facilitate future research.

Another study in a hospital in Fiji about nosocomial infections in an adult intensive care unit [16] contributed to the strengthening of infection surveillance within the facility:

*Yes, the surveillance system at the infection control department has improved.*

#### Additional documentation supporting evidence-to-policy linkage

It is always challenging to find direct linkage between evidence and policy decisions. In the present study there were some instances where additional documentary evidence was available to support that link, although it is not possible to solely attribute the policy decisions to the studies themselves.

A study by Kelly et al. in Kenya, which is an output from the 2015 OR course held in Africa, demonstrated the feasibility of using Medication Adherence Clubs for patients with chronic diseases (HIV or NCDs) in a resource-limited setting [17]. This reinforced the need to adopt a differentiated care model for patients on chronic care. The study results supported the uptake of the idea by the National AIDS programme and inclusion in the national guidelines: *A Practical Handbook for HIV Managers and Service Providers on Differentiated Care*.

Vogt et al. demonstrated a high retention rate in a decentralized community care model of HIV care through PODIs (poste de distribution communautaire), which are community-based drug refill centres to decentralize antiretroviral therapy (ART) distribution for stable patients. This evidence facilitated the adoption of the decentralized community HIV care model in the Country Operational Plan (COP) of the Democratic Republic of the Congo (DRC), 2016 [18]. There is a plan to install 10 PODIs that will be linked to high volume clinical sites (> 200 patients on ART) in 2016 which is available in the COP at http://www.pepfar.gov/documents/organization/257652.pdf [19]. The link given here is the COP of the DRC which outlines strategies to improve access and uptake of HIV prevention, care and treatment. This document strongly endorses the PODI model for improving retention under ART care and better community management of HIV+ patients. The participant shared the following:

*Now this model (PODI) is included in the national HIV policy as an innovative way to sustain ART programs. PEPFAR (The President’s Emergency Plan for AIDS Relief) has recommended to all its agencies and implementing partners to support this model in DRC. The CDC and USAID operational plan for 2016 will support the implementation of five PODIS in Kinshasa and Lubumbashi.*

This study influenced a major shift in HIV care even before the publication of the study findings. The uptake of research findings started in 2016, as mentioned in the COP of the DRC, whereas the study findings were published in 2017 [18].

Another study from Belarus on TB among healthcare workers supported a change in the national policy [20]. It was decided by the Ministry of Health that all TB cases among medical workers should be represented on the Republican TB Consilium (Board of TB experts). The participant made a reference to the official letter from the Ministry of Health Board in this regard: (Decision of the Board of MoH No. 28.3, 26.11.2014, p. 2.3).

#### Reported lack of policy and/or practice change

A total of 17 participants out of 65 had reported no impact on policy and/or practice. Of these, seven did not mention a reason and the remaining 10 described their reasons which were classified into the following themes: ‘need more time to demonstrate any such impact’; ‘study not yet or recently published’; ‘left the job and moved out’; and ‘policy change already happened, unrelated to the study’. Another five participants responded to the question on impact of research on policy and/or practice saying ‘yes’; however, their arguments were not convincing and their studies were deemed to have no influence on policy and/or practice. These decisions were made after obtaining further clarifications from the participants. Wherever required, the senior facilitators and local stakeholders were contacted via Skype/e-mail/telephone to confirm the facts presented by the participant and to gain some information about the context in which the change happened.

A participant from India on being asked the reason for not being able to influence change in policy and/or practice, said:

*I have lost the rapport with State TB Cell due to certain issues and challenges; another reason was that I have completed MPH studies and have moved back to my native state.*

Another study from South Africa advocated that all patients with rifampicin-resistant TB–HIV co-infection should be initiated on ART regardless of CD4 count [21]. However, there had been a national policy change to start ‘all TB–HIV co-infected on ART regardless of CD4 count’ and this happened before the study was completed.

Some of the participants cited ‘manuscript not yet published/recently published/still under consideration’ as the reason for lack of policy/practice change. However, there have been instances where crucial policy decisions have been made even before the publication of the findings [18,22]. (Table 2)

## Discussion

In this study, two-thirds of the research which was completed and submitted for publication under a structured OR and capacity building programme was associated with reported changes in policy and/or practice more-or-less similar to those from two previous publications [5,6]. This is encouraging as it shows that SORT IT continues to impact policy and practice after embracing a decentralized regional and national focus. We followed the journey of each participant from course enrolment to completion to the effect on policy and/or practice. Nine out of every 10 participants completed the course (i.e. submitted the manuscript to a peer-reviewed journal) and an equal proportion among those submitted have been published already. These outputs are quite impressive considering that the majority of participants were programme implementers with little or no prior research experience. These outputs are in stark contrast to another study which assessed all participants attending a similar capacity building course between 2001–2007 at the Research Institute of Tuberculosis in Japan. The study found that less than 40% of enrolled participants started a research project and none published a scientific paper [23]. Besides impact on policy and/or practice, previous studies have also shown that a significant proportion of participants who completed SORT IT training courses continue to engage in OR projects and publish papers [24,25]. This provides encouraging evidence of the long-term value and self-sustainability of this capacity building model. This paper outlines the impact of research on policy and/or practice which is seldom reported by researchers, thus adding to the limited body of knowledge in the literature and strengthening the reproducibility and consistency of evidence.

A study by Morris et al. has reported a lag of 17 years for research to impact clinical practice, with some public health research evidence even requiring more than half a decade to impact policy [26,27]. In contrast, we have reported effect on policy and/or practice within 2–3 years of the completion of the project.

In this study, we did not explore the specific reasons why the research undertaken contributed to changes in policy and practice. Indeed, policy and/or practice change is a complex phenomenon as various factors could contribute to change. While it is practically difficult to decipher the contribution of specific factors, we speculate on the following reasons for this positive linkage: (1) some participants were working in programme settings in close liaison with the key policy stakeholders that enhanced their direct role in fostering change; (2) the identification of programme relevant research questions right at the start; and (3) early engagement of policy makers and programme managers from the stage of formulation of the research question until it was published [28,29]. These are favourable factors in englobing research results within a conducive environment for fostering absorption of study findings and eventual contribution towards policy/and or practice change.

We acknowledge that a single paper is unlikely to change policy and/or practice by itself, although we do not rule this out completely. Strong evidence from a multi-centric study involving multiple stakeholders might well bring about a policy change by itself [30]. However, it is possible that, in a given context with favourable background evidence, a single study may precipitate the final decision for policy change. But generally speaking, policy and/or practice change is a complex process involving many players and should take all the available evidence into consideration. Thus, each piece of evidence could contribute towards the process of policy making. But the challenge here has been to find documentary evidence linking the research to policy decisions which requires more robust methodology, in-depth assessment and independent validation of the reported change with all concerned stakeholders. This calls for further research.

Another challenge that we encountered was deciding whether the research contributed to policy or practice changes. For example, the study by Kyaw et al. in Myanmar contributed to the decision of adopting an alternative low cost screening method (urine protein testing) by the national programme for screening of renal dysfunction in patients taking tenofovir. A detailed understanding of the existing policy guidelines and the programmatic context would be required to answer whether policy or practice changed in this situation; perhaps both. Therefore, we have presented the results in terms of any change in ‘policy and/or practice’ rather than disaggregating them. Again, there were some studies which contributed to the scale up of the existing interventions without actually fitting into any of the two categories mentioned above.

### Strengths and limitations

Two authors (JPT, AK) analysed the content and generated themes. Another two senior authors (ADH, RZ) were consulted for resolution of any disagreement. This helped minimize subjective interpretation of responses. The primary author/any other co-author, including the local co-authors who were involved in the study, were also contacted if any clarification was needed. The major limitation in this study was the self-reporting of change in policy and/or practice by the authors of the papers. As the respondents themselves were the primary investigators of the study, the self-reporting of a favourable change due to social desirability bias is a concern. Although we also tried to verify the responses by asking for more clarification from the authors, or by going through official policy documents/notifications/minutes of meetings/government orders, responder bias cannot be ruled out. A conservative approach was adopted in assessing policy impact. When in doubt, a study was deemed not to have had any impact on policy/practice. In addition to self-reporting we are currently working on developing an independent framework for impact evaluations that should improve the overall objectivity of future evaluations of evidence to policy. Further, the reasons as to why the research undertaken led to policy and/or practice change could not be systematically explored in this study.

Another limitation was that many participants did not give reasons as to why their study had no influence on policy and/or practice, and this is an area that merits further evaluation.

Despite these limitations, the study provides useful insights on the long-term impact of the research conducted in the decentralized SORT IT courses. There are few published studies looking at the impact of research conducted as part of a capacity building programme on policy and/or practice [5,6]. Although two-thirds of the studies were associated with changes in policy and/or practice, one-third was not. There is no defined threshold or standard to assess if this is high enough, but since policy and/or practice change is often a complex process we feel this is acceptable. Furthermore, it is difficult to predict at the outset which studies may be able to influence policy and/or practice. Keeping a keen eye for relevant research questions that have a greater likelihood of influencing change and engaging policy makers early may increase ‘value for money’ invested in SORT IT.

## Conclusion

This study shows that nearly two-thirds of SORT IT course papers contributed to a change in policy and/or practice as reported by the participants. This occurred while SORT IT moved to new geographic areas with a regional or country-level focus. This model may be considered for replication elsewhere, especially in LMICs where policy making is often not informed by evidence [35]. However, identifying the actual research to policy/practice linkage is challenging and requires more robust methodology, in-depth assessment and independent validation of the reported change with all concerned stakeholders.

## Paper context

The Structured Operational Research and Training Initiative (SORT IT) is a successful model of integrated operational research and capacity building. The aim of the SORT IT programme is to teach the practical skills of conducting and publishing research and influence policy and/or practice. The study aims at assessing the influence of research papers from six SORT IT courses conducted between April 2014 and January 2015 on policy and/or practice.
